# Is the suckling period and application pattern relevant for fluazuron against tick infestation in cows and their suckling calves?

**DOI:** 10.1186/s12917-021-03090-7

**Published:** 2021-12-06

**Authors:** Gonzalo Suárez, Diego Robaina, Agustina Muela, Saporiti Tatiana, Florencia Puigvert, Silvana Alvariza, Lucia Pareja

**Affiliations:** 1 Unidad de Farmacología y Terapéutica, Departamento Hospital y Clínicas Veterinarias, Facultad de Veterinaria, Universidad de la República, Montevideo, Uruguay; 2 Grupo de Análisis de Compuestos Traza, Departamento de Química del Litoral, Cenur Litoral Norte, Universidad de la República, EEMAC, Ruta 3 Km 363, 60000 Paysandú, Uruguay; 3 Unidad de bioquímica, Departamento de Biociencias, Facultad de Veterinaria, Universidad de la República, Montevideo, Uruguay

**Keywords:** Cattle, Benzoylphenyl ureas, Milk, Residues, *Rhipicephalus microplus*

## Abstract

**Background:**

Fluazuron is a chitin synthesis inhibitor administered as a pour-on formulation in cattle for tick control. This study analyzes under endemic tick infestation, the incidence of the pour-on application pattern on the plasma levels of fluazuron in calves and cows in the lactation period of the beef cow. Two hundred and ninety-two beef cows around parturition were treated with a commercial pour-on formulation of fluazuron at a rate of 2.5 mg/kg of body weight. A total of 4 treatments were carried out on days 0, 32, 77, and 117. At each administration time, the cows were grouped according to the pour-on administration pattern: long (~ 60 cm pour-on application surface) and short (~ 30 cm pour-on application surface). Fluazuron levels in cows and calves plasma were determined before the third and fourth application for each subgroup (*n* = 10) by HPLC-MS/MS. During the entire study, cow-calf pairs were maintained under field conditions and qualitatively examined for tick infestation on the day of each treatment. Both treatments (long and short) schemes were designed to prevent the annual persistence of ticks.

**Results:**

No animals with presence of ticks were identified during the first 117 days of the study, except for three cows and one calf at the time of the third application (day 77). There were no differences after 40 days (day 77) post-treatment of the second application (30 ± 5 ppb vs. 28.5 ± 12 ppb, *p* > 0.05) and 45 days (day 117) after the third application (147 ± 55 ppb vs 140 ± 46 ppb, *p* > 0.05) between groups of cows treated with the long or short pour-on application, respectively. Plasma concentration of fluazuron at second and third application was increased (3.3 and 2.9 times, respectively) in calves under free suckling compared to cows. Nevertheless, both groups of cows and calves showed a significant increase in plasma concentration of fluazuron between times (4.9 times, *p* < 0.0001 and 2.8 times, *p* < 0.0001, respectively). In both groups, tick prevalence was 0% throughout the trial, except for day 77, which reached 1%.

**Conclusions:**

The main conclusions of this study were the following: 1) Different administration patterns (long vs. short) did not differ in plasma levels of fluazuron.; 2) Given that only the cows were treated and lactating calves presented higher plasma levels of fluazuron than cows, passage through milk appears to be relevant and possibly due to a cumulative effect and continuous drug intake.

## Background

Tick infestations constitute one of the major problems in livestock production in Uruguay and South America [1, 2]. The use of acaricides in Uruguay has been the main tool for tick control [3]. In control programs of ticks, drug administration requires a strategic dosage plan with established times according to the residuality of the drugs or depending on the establishment’s tick control or eradication program [4]. In tick control programs, dip acaricides must be used with organophosphates, pyrethroids, and amidines, while with injectable and pour-on applications, macrocyclic lactones, fipronil, and fluazuron are the most relevant [5]. Resistance has been reported for all the aforementioned drugs, except for fluazuron, which has no practical in-vitro tests for its detection [6].

Fluazuron is responsible for inhibiting chitin synthesis on immature ticks, preventing them from achieving adult stages. Insect growth regulators, such as fluazuron, could be responsible for the long-term response given the delayed effect that this type of compounds have on insect’s life cycle [7], reducing the number of immature life stages of *Rhipicephalus microplus* (*R. microplus*).

The application of pour-on formulations of fluazuron in the initial period of lactation generates uncertainty about the levels reached in cows or their lactating calves. Likewise, the persistence levels or the elimination rate of fluazuron in lactating cow beef are not clear [8]. Concentration levels due to digestive absorption in lactating calves are also not reported. Furthermore, differences in the practical application technique at the field level, using different empirical recommendations in the topical administration of the product, varying the extension on the application surface, but maintaining the dose, remains uncertain.

Therefore, the efficacy and associated benefit of the fluazuron poured preparation as a tick control alternative in lactating cows needs to be documented. The objective of this study was to determine, in endemic tick infestation, the incidence of the application pattern of fluazuron pour-on on the plasma levels in cows and calves in the lactation period of the beef cow.

## Results

The prevalence of ticks at the beginning of the experiment (day 0) was 1% (3/292), as expected by the epidemiological model described for Uruguay. In the following evaluations (32, 77, and 117 days), no animals with presence of ticks were identified, except for three (in short group 0.7% [1/145] and in long group 1.3% [2/147]; *p* > 0.05) and one calf at the time of the third application (day 77). The infestation rate was not different (Odds ratio 0.5, CI_95%_ [0.008,9.79]) between the two application surfaces groups throughout the evaluation periods.

**Fig. 1:**
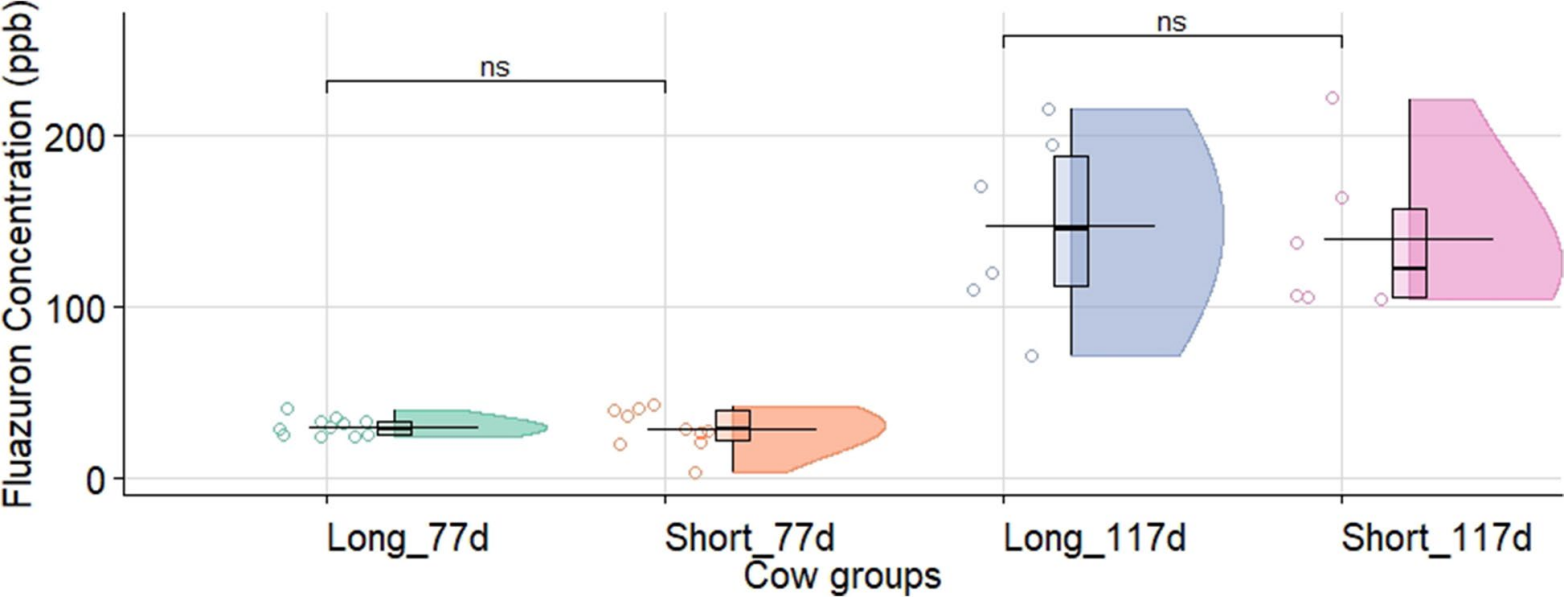
Fluazuron cow plasma concentration (ppb) before dose repetition (77 and 117 days from the first dose) of a fluazuron pour-on formulation (2.5 mg/kg, pour-on, 2.5%, Acatak®), following two different application schemes: *short* (30 cm) and *long* (60 cm). Legend: Long_77d = long application at day 77; Short_77d = short application at day 77; Long_117d = long application at day 117; Short_117d = short application at day 117. *P* Value Pairwise (T-test) between groups (ns: *p* > 0.05). The concentration (ppb) are summary statistics in the box-plot type (interquartile range) and half-violin plot. The horizontal line shows the mean concentration

**Fig. 2:**
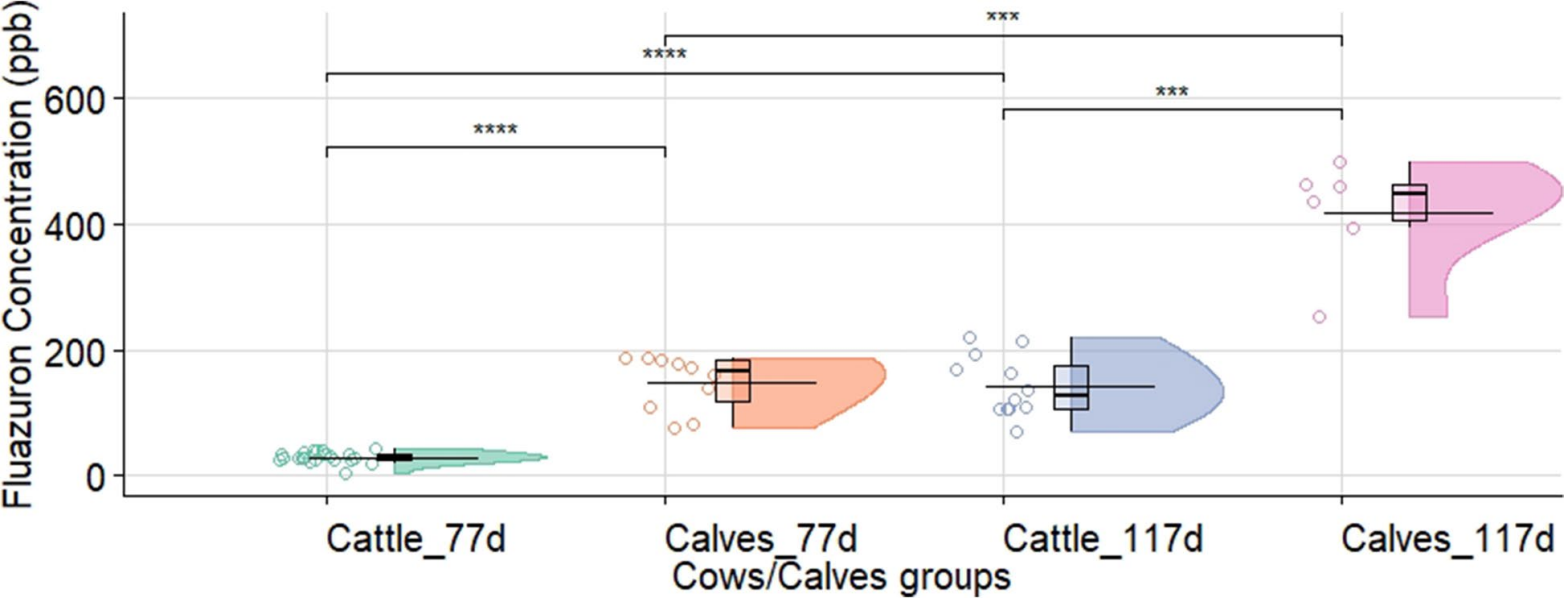
Fluazuron cows and calves plasma concentration (ppb) before dose repetition in cow (77 and 117 days from the first dose) of a Fluazuron pour-on formulation (2.5 mg/kg, pour-on, 2.5%, Acatak®). Legend: Cattle_77d = cow at day 77; Calves_77d = calves at day 77; Cattle_117d = cow at day 117; Calves_117d = calves at day 117. *P* Value Pairwise (T-test) between groups (***: *p* < 0.001; ****: *p* < 0.0001). The concentration (ppb) are summary statistics in the box-plot type (interquartile range) and half-violin plot. The horizontal line shows the mean concentration

The individual levels of fluazuron in cows determined prior to the third and fourth repeated application for each subgroup (*n = 10*) are presented in Fig. 1. There were no differences after 40 days (day 77) post-treatment of the second application (30 ± 5 ppb vs 28.5 ± 12 ppb, *P* > 0.05) or at 45 days (day 117) after the third application (147 ± 55 ppb vs 140 ± 46 ppb, *P* > 0.05) between the groups of cows treated with the long or short pour-on application, respectively. In view that the cow groups (long vs short) did not present statistical differences, it was all considered as a global cow group. Forty days after the second administration (day 77), the average concentration (± standard deviation) for the global cow group and calves were 29.3 ± 8.9 ppb and 147 ± 43 ppb (*P* < 0.0001), respectively (Fig. 2). The application of the third repeated treatment and the determination of the concentrations 45 days later (day 117) increased the average concentration reaching 143 ± 49 ppb and 417 ± 87 ppb (*P* < 0.001) in the treatments of global cow and calf groups, respectively (Fig. 2). Plasma concentration of fluazuron prior to the third and fourth application increased 3.3 and 2.9 times (respectively) in calves under free suckling vs. cows (Fig. 2). Nevertheless, the global cow group (4.9 times; *P* < 0.0001) and the calves (2.8 times; *P* < 0.001) showed a significant increase in plasma concentration of fluazuron, between 77 at 117 days (Fig. 2).

## Discussion

The bioavailability of a drug is directly dependent on its rate and degree of absorption at the administration site. Factors that affect the absorption of the drug, including patterns for the pour-on application, will directly affect the bioavailability of the drug. Our results suggest that the drug dispersion pattern long vs. short does not represent a relevant variable when the correct dose is administered. The topical absorption of the pour-on formulation can be explained through the Fick law of diffusion, which established that the drug molecules move according to the concentration gradient from a higher drug concentration to a lower one until equilibrium is reached [9]. Although in our study we doubled the absorption surface (line of application pass the 30 cm to 60 cm), we did not modify the dose, therefore the concentration was decreased per contact surface, which possibly determined that the passive diffusion of the drug was not significantly modified.

When redosing lipophilic drugs such as fluazuron at established times, not only the pharmacokinetic profile on the dam is affected, but also the plasma levels on lactating calves. After pour-on treatment at 1.5 mg/kg, the mean plasma levels remained stable between 9 and 35 days after treatment, ranging from 35 to 41 μg/L and declined at about 7 μg/L at 16 weeks [10]. Using the same dose and formulation, but from a single administration and during a short period, Ferreira et al. [11] reported lower plasma concentration values. In our study, the dosing interval was set at 4–6 weeks following the manufacturer recommendations and the governmental guidelines on tick control for Uruguay, which could explain the higher concentration levels of fluazuron found in cows and calves. In addition, we used a dose of 2.5 mg/kg as recommended by the manufacturer. Both the dosing period and the higher dose could explain the higher plasma levels for fluazuron found in our study. The Committee for Medical Products for Veterinary Use [12] issued a Summary report on fluazuron pharmacokinetic behavior, with fluazuron being excreted via cow’s milk to calves, resulting in higher plasma and fat residue levels in calves compared to the ones in cows.

Accumulation is usually considered on plasma, overlooking the effect on tissues with poor blood perfusion [13], leaving the explanation for drug accumulation on lactating animals relegated as to whether accumulation occurs and the possible impacts of such process. According to [12], a steady state between absorption and elimination was observed for 3 to 4 weeks after treatment. When a single dose of fluazuron is administered topically to cattle, the depletion from plasma is slow, with an elimination half-life of 10.5 weeks. Therefore, multiple doses could lead to drug accumulation both in the cow and the calves. As reported by [12], multiple treatments with 12-week intervals did not lead to the accumulation of fluazuron residues.

The greater accumulation of this acaricide in calves concerning lactating cows and the increase between subsequent administrations, could be explained by the continuous intake via the digestive tract through milk. Different authors document the importance of licking in topical administrations and the relative importance of the dermal and oral routes in the removal of drugs from the skin [14, 15]. We ruled out the licking effect since at the time of application we made a quick visual inspection and determined a null licking behavior in the first hours after applying all treatments (Unpublished Data). As stated by [16], the excretion of parent compounds and/or their metabolites from plasma into milk is a complex process related to physicochemical properties and membrane interactions. Fat content is one of the main factors that contribute to the concentration of hydrophobic drugs into milk [17, 18]. Given the lipophilic characteristic of fluazuron, fat and milk residue levels are expected to be higher than in plasma.

In this study, the prevalence and burden of *R. microplus* found in the animals are consistent with the data provided by the conceptual epidemiological model described by [1] for Uruguay. Under ideal temperature and relative humidity conditions (27 °C and over 80% RH), the first generation of ticks takes place between August and October, while the second generation appears from December to February. The tick populations parasitizing cattle is higher in the first generation, with an average of 25 ticks per animal. Considering that the study was carried out on a farm with a background of tick infestations in previous autumns and that the prevalence was lower than expected by the epidemiological model, we can consider that the concentration levels of fluazuron in the animals were enough to prevent the development of the parasitic cycle of the tick.

The rational use of drugs is crucial in food-producing animals because of the potential adverse effects of drugs that appear as residues in edible tissues [19–21]. Milk excretion needs to be quantified in order to understand the pharmacokinetic changes that could occur during the suckling period and the impact on parasites control and drug residue levels, especially on parasites with high health impact such as *R. microplus*. Further studies are needed to quantify milk excretion of fluazuron under pour-on administration on lactating cows. Achieving a pharmacokinetic model for this excretion route is the first step in understanding the use of this chemical tool for tick control around parturition.

## Conclusions

The main conclusions of this study were the following: 1) Different administration patterns (long vs. short) did not differ in plasma levels of fluazuron; 2) Given that only cows were treated and lactating calves presented higher plasma levels of fluazuron than cows, passage through milk appears to be relevant and possibly due to a cumulative effect and continued drug intake.

## Methods

### Study location

The study was conducted from August to December 2019 (end of winter and beginning of summer) on a farm located in Artigas, Uruguay, South America (30°06′ South, 57°04′West). The farm has a history of tick and use of all approved drugs (Ivermectin, Fipronil, Amitraz, or Fluazuron) for the control of *R. microplus*. Animals were not treated with acaricides in the 60 days previous to the fluazuron treatment. Tick infestations were present in a very low number of animals on the farm prior to the beginning of the study. The prevalence was as expected for the period selected for the trial, corresponding to the first generation of ticks within the epidemiological model of the country [1]. Rainfall during 2019 (January to December) was 1440 mm, and the minimum and maximum temperatures were 4.6 °C (July) and 30.9 °C (October), respectively.

### Experimental design

Two hundred and ninety-two beef cows (Hereford) around parturition were treated with a commercial pour-on formulation of fluazuron (fluazuron 2.5%, pour-on, Acatak Pour-on®, Novartis, Brazil) at a rate of 2.5 mg/kg of body weight. A total of 4 treatments were carried out with a dosing period set in a range of 32 to 45 days. At the administration time, two different patterns for the pour-on application were randomly tested and used as a grouping variable: *long* (~ 60 cm pour-on application surface according to the recommendations on the label of the surface to be covered by the product, *n* = 147) and *short* (~ 30 cm pour-on application surface [half surface], *n* = 145). The calves remained free of antiparasitic treatment. Throughout the study, cow-calf pairs were kept in the same paddocks and under field conditions (native grasses). Both treatment schemes were designed to prevent the annual persistence of ticks. A control group was not included because the farm was in a global tick control strategy.

### Data recovery

### Quantitative analysis of tick infestation:

All the cows and calves in the study were qualitatively examined for tick infestation on the day of each treatment (day 0, 32, 77 and 117). Each animal was examined for the presence of ticks (adult engorged female ticks; 4.5–5 mm of diameter) on the head, ears, neck, back, perineum, and tail [22]. A careful examination was carried out in 10% of the animals (randomly selected), which included the examination of the belly and legs. Tick presence was recorded as a binary variable (presence/absence of ticks). An animal showing one or more ticks was considered a positive case (presence), while animals free of ticks were considered as negative cases (absence).

### Fluazuron analytical procedures

Before the third and fourth application (days 77 and 117, respectively), blood was taken from 10 animals from each group of cows (*long* and *short*) and 10 calves randomly (using heparinized tubes).

Fluazuron concentration in plasma was determined by HPLC-MS/MS, based on the procedure validated by our group with acceptable accuracy according to the guidance document on analytical quality control and method validation procedures for pesticide residues analysis in food and feed from the European Commission [23]. Matrix-matched calibration curves in the range from 5 to 1000 ppb (μg/kg) were established using least-squares linear regression analysis and correlation coefficients (*r* = 0.99) and back-calculated concentration residuals below 20%. A limit of quantification (LOQ) of 5 ppb was defined as the lowest measured concentration with a relative standard deviation below 20% and an absolute recovery ≥70%. Concentration values below the LOQ were not considered in the analysis of experimental data.

### Statistical analysis

The prevalence (%) of ticks was determined as the ratio of the number of existing cases during a certain period of time and the population at risk during that specified period [24]. The plasma concentration data are reported as arithmetic mean ± SD. Differences between cows groups (*long* or *short*) at each time were analyzed for statistical significance using the independent samples t-test. Since the long and short groups did not present statistical differences, they were considered a single group of cows (global cows group). The differences between calves and the global cows group within and between times were analyzed for statistical significance using the independent samples t-test, previously applied Shapiro-Wilks test and graphical method to check normality. Statistical analyses of differences in the prevalence rates between groups were performed with Fisher’s exact test. A *p*-value < 0.05 was considered statistically significant. The statistical analysis was performed using R Statistical Software (version 4.0.3 [2020-10-10]) [25].

## Data Availability

The datasets used and/or analyzed during the current study are available from the corresponding author on reasonable request.

## Abbreviation

HPLC-MS/MS: High Performance Liquid Chromatography Tandem Mass Spectrometry
